# Barriers and Facilitators of Communication in the Medication Reconciliation Process during Hospital Discharge: Primary Healthcare Professionals’ Perspectives

**DOI:** 10.3390/healthcare11101495

**Published:** 2023-05-21

**Authors:** María Jesús Rojas-Ocaña, Cristina Teresa-Morales, Juan Diego Ramos-Pichardo, Miriam Araujo-Hernández

**Affiliations:** Department of Nursing, University of Huelva, 21071 Huelva, Spain

**Keywords:** medication reconciliation, assistance levels, safety communication, medication errors

## Abstract

The WHO established that medication errors are the most common and preventable errors and represent an expenditure of 42 billion U.S. dollars annually. The risk of medication errors increases in transitions between levels of care, mainly from hospital care to primary healthcare after hospital discharge. In this context, communication is a key element in the safety of the medication reconciliation process. The aim of this paper was to describe the barriers to, and facilitators of, effective communication during the medication reconciliation process at hospital discharge in people over 65 years of age, from the perspective of primary healthcare professionals. A qualitative descriptive study was designed, and in-depth interviews were conducted with 21 individuals, of whom 13 were nurses and 8 were physicians. This study was carried out with healthcare professionals belonging to primary healthcare centres in Huelva (Spain). Following content analysis of the discourses we identified 19 categories, grouped into three areas: interlevel communication, communication between primary healthcare professionals, and communication between healthcare professionals and patients/caregivers. The barriers found mainly relate to the adequacy and use of technological tools, time available, workload and the level of collaboration of patients/caregivers. Facilitating elements for communication in medication reconciliation included technologies, such as computerized medical history, protocolization of clinical sessions, the presence of case management nurse and interdisciplinary teamwork.

## 1. Introduction

The National Coordinating Council for Medication Error Reporting and Prevention define medication errors as ‘any preventable event that may cause or lead to inappropriate medication use or patient harm’ (about Medication Errors|NCC MERP). The WHO has established that, globally, medical errors are the most common and preventable healthcare miscalculations, and they represent an expenditure of 42 billion U.S. dollars annually [1]. For this reason, halving the number of drug incidents is the goal of the WHO’s third patient safety challenge [1], as drug safety is an indicator of the quality of care and patient safety [2,3].

However, only the developed countries are equipped to allocate a substantial budget to reduce medical errors, because globally, not all countries share equal access to medicines and healthcare finance [4]. Particularly, in rural and suburban areas of low and middle-income countries, people are still frequently denied access to even the most fundamental generic pharmaceuticals. These difficulties are worsened by the lack of evidence-based resource allocation strategies and unsustainable financing strategies [4,5].

Personal conditions, such as advanced age and the presence of chronic diseases, comorbidities and polypharmacy, defined as a situation in which the patient uses five or more medications at once [6], increase the risk of medical errors [7]. In itself, polypharmacy is a criterion of frailty in older adults and a risk factor for mortality and morbidity as it increases the risk of drug interactions, increases the risk of anticholinergic and sedative burden [8], decreases therapeutic adherence and demands greater use of health resources [7,9]. Numerous studies have also argued that transitions between levels of care are a source of discrepancies in medication, medical errors and potentially adverse events [10,11,12], occurring mainly in the transition from hospital care to primary healthcare, after hospital discharge [10,13,14,15,16]. The WHO states that more than 40% of medical errors occur in the transition from hospital to home [1]. These errors may result from a lack of reconciliation between the previously prescribed medication and the new one, as well as unintentional omissions in previous medications, duplications and other errors [11,17,18]. Thus, people over 65 years of age with chronic disease and patients with polypharmacy who are transferred from hospital to primary health centre seem to be the most vulnerable to potential medical errors.

Medication reconciliation is defined as the formal, standardized process of obtaining the complete list of a patient’s previous medication, comparing it to active prescribing, and analysing and resolving any discrepancies found [19]. This practice has been proposed as one of the main strategies to reduce discrepancies and medical errors, as well as to ensure patient safety, especially after hospital discharge [15]. Successful medication reconciliation not only prevents medical errors, but also reduces the rate of readmissions and increases patient safety [10,16,20].

In addition, the patient’s allergies and their previous history of adverse events in relation to the medication should be included to constitute a multidisciplinary network that also involves patients and their informal caregivers as well in the process of medication reconciliation [10,16,20,21,22].

Previous studies have shown that difficulties in carrying out effective medication reconciliation are mainly related to collaboration and communication between healthcare professionals [23]. The system used in the Andalusian Health Service to support the electronic medical record is Diraya^®^, which integrates all the health-related information of the people treated in the health centres, ensuring their availability at the right time and place whenever required to treat the patients and also ensuring efficient management of the health system (Diraya|Servicio Andaluz de Salud (juntadeandalucia.es)).

The difficulties in the medication reconciliation process have been related to care overload and lack of time [3,13,21], lack of training, knowledge and skills of healthcare professionals [17,21], lack of standardization in the medication reconciliation process [22,24] and the dissolution of medication reconciliation responsibility among healthcare professionals due to ambiguity over who should assume responsibility and execute it [17,24].

The main actors in the medication reconciliation process are the patients and/or their informal caregiver and all the healthcare professionals. In Andalusian Health Service, these professionals are nurses and physicians of the hospital and of the primary healthcare centres. During the discharge process in the Andalusian Health Service, the case management nurses is identified as the link between patients/caregivers, healthcare professionals [25].

Poor multidisciplinary communication with the hospital and fragmented communication between levels of care lead to chaotic and non-systematic transitions [21,26]. The need to improve communication between healthcare professionals and patients and their informal caregivers has been shown to ensure proper medication reconciliation [11,22,27,28]. Taking such facts into account, the WHO has stated that good communication is vital for successful medication reconciliation, so it proposed to create and implement a specific communication strategy for it [1].

Therefore, initiatives that aim to improve quality and guarantee the safety of transitional care must address system fragmentation, ensure adequate professional training, and reduce communication barriers within and between healthcare settings [16,17,21,23,29]. Thus, identifying the factors that influence communication from the perspective of the healthcare professionals involved, and between professionals and patients/informal caregivers, so that medication reconciliation is carried out with all the possible safety guarantees, is of paramount importance. Thus, the objective of this study was to describe the barriers to, and the facilitators of, effective communication in the medication reconciliation process at hospital discharge in people over 65 years of age from the perspective of primary healthcare professionals.

## 2. Materials and Method

A descriptive qualitative study, adhering to the COREQ guidelines [30], was designed to achieve our aims. The dimensions were barriers to, and facilitators of, effective communication in the medication reconciliation process.

### 2.1. Setting and Participants

This study was carried out in the city and province of Huelva (Spain), where the Andalusian Health Service guarantees universal, equitable and free care to the entire population on two levels: hospital care and primary healthcare [31]. There are two types of centres that provide primary healthcare: urban centres catering to a population of at least 12,000 inhabitants who reside in the same town; and rural centres, for populations less than 12,000 inhabitants who reside in the same town or nearby [32]. Primary healthcare centres have a multidisciplinary team of workers, including nurses, case management nurse, social workers, physicians, and midwives. There are 29 primary healthcare centres in the province of Huelva; 18 urban (62%) and 11 rural (38%), with 1.07 nurses and 0.8 physicians per 1000 inhabitants (Information by centres|Andalusian Health Service—Junta de Andalucia).

With this setting in mind, we designed an intentional selection strategy to include physicians and nurses who worked in the urban and rural primary healthcare centres. The purpose was to select the key participants who had faced medication reconciliation-related situations in patients over 65 years of age discharged after hospital treatment. Thus, we proposed a single inclusion criterion: all participants must have had at least two years of experience in professional healthcare practice.

We explained the aims of this study to the managers of the primary healthcare centres and asked them to disseminate this information among the groups established so that the individuals offer their candidature to partake in our study. Those interested in participating were contacted by telephone by the researchers who, after confirming their willingness to participate, invited them to an in-depth interview. Applying the snowball technique [33], new participants were contacted progressively until reaching the information saturation point [34].

### 2.2. Data Collection

This was done through individual, face-to-face, and in-depth interviews conducted by three experienced researchers who applied the following topic script:

Sociodemographic: sex and age.

Work conditions: profession; work centre and area; years of experience; and specific training in medication reconciliation.

Perceptions of medication reconciliation at hospital discharge in people over 65 years of age: information received at the time of patient discharge; procedure between patient discharge and new consultation at primary healthcare; professionals who participated in the process; and coordination between care levels.

Facilitating and hindering elements of communication in relation to medication reconciliation at discharge: pathways by which they receive information after discharge; protocols they have/follow for the incorporation of new medication reconciliation information; communication technology tools; communication strategies in MR; strengths and weaknesses of the process; and interaction with patients/informal caregivers.

### 2.3. Data Analysis

Content analysis of the participants’ discourses was carried out using the Graneheim and Lundam approach [35]. This analysis technique involves studying the answers of the participants until the identification of the units of meaning for their subsequent codification and categorization into topics that answer the research question [35]. The recordings of the interviews were visualised and transcribed verbatim. The researchers independently completed an iterative process to become familiar with the data through successive readings and re-readings of the speech. They identified the units of meaning related to communication barriers and difficulties in the medication reconciliation process. The units of meaning were coded based on their content, and the codes generated were compared, unified and reduced according to their similarities, and grouped into categories based on their similarities of content and meaning. The categories were subsequently classified into three areas of communication that the literature had shown as relevant: interlevel care; among primary healthcare professionals; and between primary healthcare professionals and patients/informal caregivers.

This process required several meetings to discuss, agree, rename, unify and reduce codes and categories by reflective consensus.

Finally, we analyse the interactions between codes through cognitive networks extracted from the co-occurrence analysis objective criteria for categorization and for establishing the relationship between categories. This analysis allows to know the absolute frequency of citations (context units) where the code of rows and columns coincide; and the coefficient of co-occurrence, that is, the number and link of the citations where they coincide in relation to the total citations of both codes. In this case, the links in the collective discourse with those codes whose coefficient of co-occurrence equalled or exceeded 0.1 were considered. On the other hand, the qualitative criterion was valued based on the emphasis of the content of the citations, that is, two codes were considered linked when they occurred in a unit of meaning and presented a significant, symbolic or interesting link with the objectives of the research. For the analysis process, the Atlas.ti program was used.

### 2.4. Trustworthiness

Trustworthiness was established using Lincoln and Guba trustworthiness criteria [36]. To ensure the credibility, dependability, confirmability, transferability and authenticity of the data, the following strategies were employed. Two experienced researchers conducted the interviews. The analysis of the interviews and the coding process were performed independently by different researchers. Several meetings were held during the codification and categorization process to agree, unify and limit codes, and to categorise the topics. At the end of the analysis, the participants were invited to join a discussion group, attended by six of them, wherein the results of the analysis were announced, after which the informants expressed their agreement with what was stated, thus ratifying the results of the analysis.

### 2.5. Ethical Considerations

The study was conducted in compliance with the ethical and legal standards derived from the Declaration of Helsinki, and was approved by the Andalusian committee for ethical research in health (ref: PPPCM/21, with date 11 May 2020).

## 3. Results

### 3.1. Participants’ Characteristics

A total of 21 individuals participated in this study, 13 nurses (ID: N.1–N.13) and 8 physicians (ID: P.1–P.8). Of them, 62% worked in urban centres (seven nurses and six physicians) and 38% worked in rural centres (six nurses and two physicians). Additionally, of the total participants, thirteen were women (ten nurses and three physicians) and eight were men (three nurses and five physicians). The mean age of the participants was 50.57 years.

The healthcare professionals who participated in this study had an average work experience of 24.14 years, with the shortest time of 6 years and longest of 38 years. All participants met the inclusion criterion, having at least two years of experience in primary healthcare, with the average of working in this sector being 15.59 years.

### 3.2. Descriptive Analysis

The analysis of the arguments of the interviewees generated 90 units of meaning, which were coded into 65 codes, categorized into 19 categories that were reviewed and grouped into 3 areas referred to in the literature as relevant: interlevel communication (8 categories); communication between healthcare professionals involved in medication reconciliation in primary healthcare (4 categories); and communication between healthcare professionals and patients/informal caregivers (7 categories).

**Figure 1:**
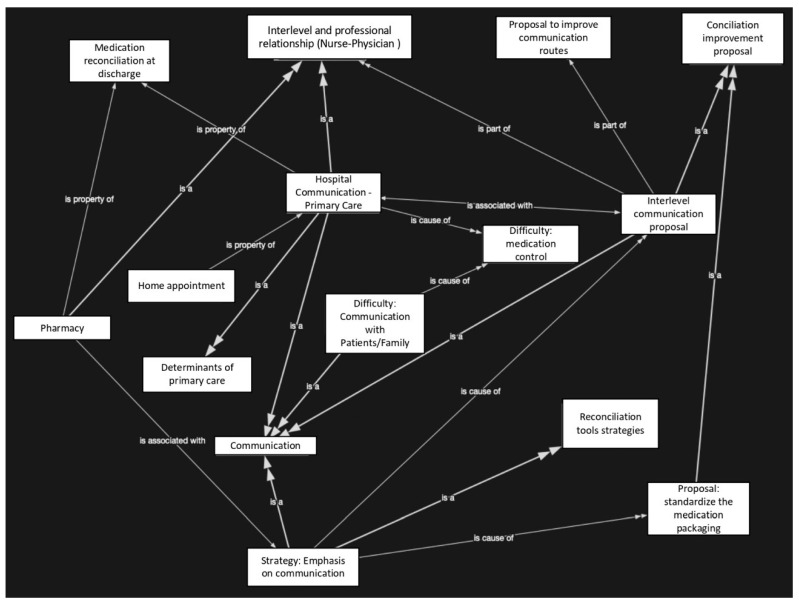
Cognitive network of relationship of communication codes.

The code cloud (Figure 1) shows the interactions established between the codes resulting from the analysis of the data from the interviews carried out. The image reflects the intensity of the interactions by the thickness and direction of the arrows. The arrows that have a greater thickness and that in turn have a double arrow at the end, reflect interactions of greater intensity, that is, more citations that interrelate these two codes. Unlike those that are thinner and have only a triangle at their end, they reflect a relationship, but of lower intensity. In addition, the figure presents the type of interaction they have, indicating “is a”, “is part of”, “is property of”, “is a cause of” and “is associated with” as a reflection of the relationship between both codes. This relational information, together with the direction of the arrows and their thickness, reflect greater interaction in the established dimensions between interlevel care (hospital care and primary healthcare), communication between healthcare professionals involved in medication reconciliation in primary healthcare centres (nurses and physicians) and communication between primary healthcare professionals and the patient/informal caregiver.

#### 3.2.1. Barriers to, and Facilitators of, Communication in Interlevel Care

First, the barriers and facilitators related to the interlevel care communication scenario in the hospital discharge process are presented. Our analysis yielded ten categories, of which six were barriers and four facilitators. Table 1 shows the classified categories accompanied by their operational definition.

##### Barriers

The findings refer to the **non-existence or ineffectiveness of communication channels **as the main barrier. Both nurses and physicians report having many difficulties in reconciling the medication of their patients after discharge due to the lack of an effective relationship between the different levels.

*“That is the great evil of our health system. There is no such fluidity that should exist between the specialist and primary care. It didn’t exist 30 years ago, or 10 years ago, or today. And I doubt very much that unless things change, it will ever be. You call the number you have thousands of times and there is no way to talk to the specialist”*

N.12

*“The first thing is to improve interlevel communication, especially between primary and specialized, to be more fluid, that there is not that great barrier of communication that we have had; it is true that it is currently somewhat more fluid, although that problems still exist. If it were achieved, that would allow the patients we are both treating at the same time, he from his specialty and I from a more integral medicine, to be much better treated and above all the issue of drug conciliation would be controlled”*

P.8

Another barrier detected **is the lack of collaboration between healthcare professionals in medication reconciliation process**; they report little involvement of the professionals in the process, often prescribing medication in their treatment field without assessing the rest of the treatment that the patient receives.

*“Sometimes we miss greater collaboration from specialists, we see how most of the time, after a consultation with the specialist, the patient receives the discharge report without an explanation to the patient about taking the medication, the dosage, schedules, the possible interactions with other medications, etc. All that is in our hands, the patient comes to his physician with the report so that it is he who interprets it and makes it clear”*

P.6

*“[…] greater connection between hospital and primary care, especially in communication between professionals”*

N.11

Both nursing and physicians identified **the lack of a relationship with the pharmacist** as a barrier, resulting in a disconnection between the prescription of medications and their acquisition by the patient.

*“[…] the pharmacist, if he detects something, the tool for precautionary cancellations is used, but you have to keep in mind that you see that in the mailbox, and if you have 40 patients and you have to read the mailbox while running about in your job, it may escape you, so communication between the pharmacist and the physician should be improved; it is true that there are easier centres for this than others, since some have 13 or 14 physicians and the pharmacist cannot be in contact with everyone, but there should be some mechanism that would allow us to contact the pharmacist more quickly if they detect something that we have not detected”*

P.7

*“I also have a lot of contact with pharmacists to put a kind of signal to the mailbox, to try to link them with something, for example, at the time of the shots, because we put a cup at breakfast, at noon and at dinner, etc.”*

N.7

On the other hand, **the non-comprehensive care received by patients in hospital care**, considered as a process of fragmentation by medical specialties suffered by patients in their healthcare, was identified as another communication barrier since it generates difficulties in achieving a complete medication profile of the patient.

*“The fundamental problem I observe is that the physicians at the hospital change the treatment, but they do not modify the previous treatment, so the patient comes home with double prescribed treatment and that has to be realized by the primary care physician when he is going to renew the prescription or something like that”*

N.11

*“I emphasize that if they go to the specialist and come back with two or three new drugs that are added to those they already took, it is very difficult for the patient to believe that the physician tells him not to take this or that and listens to him, to trust the primary care physician. They come from the hospital, from the specialist and that the word is law”*

P.5

The nurses detected a barrier in relation **to the use of patients/caregivers in transferring information from hospital to primary healthcare centre**. This creates difficulties for primary healthcare professionals when attempting to find the correct medication reconciliation information, since the patient or family are not experts in the field.

*“The truth is that if the patient notifies us, then we find out, and if not, to this day I cannot know, with the volume of patients I have, if this patient has been discharged or not. Either the patient contacts us because he needs a cure, or check something, or an analysis or whatever, or I don’t know about his discharge”*

N.11

Several participants related this lack of comprehensive care to the patient’s own conditions that make medication even more difficult, as they are **chronic multipathological patients**. They referred to this condition as another barrier in the communication process for medication reconciliation.

*“No, neither in those with heart failure, there is no such communication, it is more with diabetics and hypertensives. COPD is when they come to the centre for some other reason or they have a crisis, then we do that work in the emergency room and explain to them, since they do not come to the consultation unless they have a specific problem. Nor does the physician refer me to a COPD patient, it is very rare”*

N.2

*“It would be interesting if, above all, in the most fragile patients there was a more direct communication, from physician to physician, but it is true that within the health system we have, it does not seem insufficient that we learn that they have been discharged”*

P.1

##### Facilitators

A significant facilitating element was found to be **the case management nurse**. Both nurses and physicians acknowledge that the advanced practice nurse is key in communication between levels of care, and that their presence and work is essential for medication reconciliation.

*“Normally the case management nurse at the hospital alerts the case management nurse at my health centre. Then, she is the one who has to call that person in case she needs some kind of assistance from nursing, in that case she already communicates it to us”*

N.10

*“We have a case management nurse, who does exactly that, she is a liaison between the hospital and the primary healthcare centre”*

P.3

Communication **technology tools**, such as the Diraya^®^ computer program and the healthcare professional’s mailbox, were cited as fundamental elements for communication between levels that facilitate medication reconciliation. Information on the patient’s discharge, medication prescribed, procedures developed and difficulties encountered, are just some of the information that can be obtained using these tools.

*“There are cases where the liaison nurse tells us that this person has just been discharged. Apart from that, we have the Professional Mailbox that when opening the DIRAYA we see people who have entered our quota and who have been discharged from the hospital. So in certain cases of patients who are followed up at home, in those cases we contact them and we see it directly, other times they are referred to us by the liaison nurse who is the one who receives the information directly”*

N.4

*“We have a mailbox where every day we find the discharges, There, I see every day who is discharged, who enters, who goes through emergencies”*

P.3

*“We have the Professional Mailbox where we receive notifications of admissions and discharges that occur with respect to our patients. When we open the computer in the morning, a list appears with the patients who have been admitted, or have gone to the emergency room or have been discharged. In these cases I try to go into the history of that patient and see what has happened to him and the medication”*

P.2

Finally, physicians point to the telemedicine service as a facilitator of communication between healthcare professionals facilitating medication reconciliation decision-making at discharge.

*“We know that in the hospital there is an email for the communication of information about complex chronic patients, and about some help, or some information about these patients you can write it there and after a few days the specialist answers you, but this only works with internal medicine, the ideal would be to do it with the rest of the specialties, there are other patients who are not so chronic or so complex, but also sometimes you have doubts about how to manage them and if you go to the ordinary route of making an appointment, etc., there is a lot of delay in the diagnosis”*

P.4

#### 3.2.2. Barriers to, and Facilitators of, Communication among Primary Healthcare Professionals

Communication between primary healthcare nurses and physicians involved in medication reconciliation is another area addressed in the analysis of the results of this study. This analysis yielded a total of four categories, two as barriers and two as facilitators (Table 2).

##### Barriers

The first barrier detected was the non-existence, ignorance or lack of use of a standardized system that regulates and standardizes communication on medication reconciliation between the healthcare professionals involved. The lack of sharing these procedures creates biases in communication among healthcare professionals, generating difficulties in multidisciplinary work for obtaining patient medication reconciliation at discharge.

*“I don’t have a standardized system as such. When I make the home visit to perform a cure or something, I usually take the patient’s medication sheet printed in case they have any doubts and they can be resolved”*

N.11

Another element detected as a barrier is **the lack of time for communication between nurse and physician**, in terms of failing to find those spaces in which to comment on the situation of patients whose care they share, as well as to share information on difficulties, strategies or decision-making.

*“I have my own nurse and we form a team. We would have liked to have had a little more communication, we would have liked to have a few days a week to talk and have feedback on patients. Talk not only about treatments, but also about the evolution of wounds. Unfortunately it has not been possible and with the arrival of COVID well, less, so now when my nurse sees anything, he goes through the consultation or I tell him to stop by at the last minute and we talk more calmly, or we look for a little time”*

P.1

##### Facilitators

Elements that facilitate communication on medication reconciliation **include the existence of protocols that unify criteria and forms of action**. These protocols are valued as an element that facilitates communication and intervention in patients in need of medication reconciliation.

*“From here, we get going, we call him by phone, we look at the history and in the report since we have access to the clinical station of the hospital and we see what has been the evolution and the problems that have been. We ask him if he needs our help for anything, since the medication, being new, needs clarification. We do a review, especially with older patients, by telephone”*

N.3

**The close relationship established between the different healthcare professionals (nurse and physician) involved in medication reconciliation** is identified as a facilitating element of teamwork and effective communication.

*“When we formed, the team the physician who was with me previously, we had good communication, for example, if he detected a patient with altered blood glucose and diagnosed him as diabetic and gave him treatment, he immediately called me and told me that the patient had been scheduled for the next day. Just as when a diabetic patient already on time, and it goes wrong, went from oral treatment to insulin, he called me and passed it on me so that I could explain”*

N.2

*“I get along quite well with my functional unit, the nurse I work with explains perfectly to patients how they have to take medication, clears all doubts with them, without the need for them to go through me. On the other hand our relationship is very fluid, we are talking continuously, we transfer any doubts; sometimes the patient tells one thing to the nurse and another to me differently, he wants to tell the same thing, but he doesn’t, and we have to be coordinated to know what to do with him”*

P.7

#### 3.2.3. Barriers to, and Facilitators of, Communication between Primary Healthcare Professionals and Patients/Caregivers

The third and final line of argument refers to the barriers and facilitating elements of communication for medication reconciliation between primary healthcare professionals with the patient and/or the patient’s informal caregiver. The analysis of these data yielded seven categories, five barriers and two facilitators (Table 3).

##### Barriers

The barriers detected refer to the environment in which communication with the patient/informal caregiver takes place. On one hand, **the geographical distribution of the primary healthcare centre** is important, as the setting where the care work is performed influences the possibility of communicating with the patient/caregiver.

*“[…] here there are 22,000 inhabitants and there is no communication like that in the Redondela, for example, which belongs to Isla Cristina and is a small town, and there is a pharmacy and people know each other better, but in Isla Cristina, which is a rural environment, the quotas of physicians are for 1700 or so, and then it can be put at the level of an urban environment”*

P.8

**Unstable job conditions of the staff** was identified as a barrier by nurses, since healthcare professionals do not have time to get to know the patients, nor do they have the ability to follow up, making it difficult for establishing fluid and effective communication.

*“[…] They tell the physician that they have understood and then they come to you, and it happens that sometimes we have had many changes of physician, few stable staff; the stable staff were the nurses and they came to us to ask us, since they were in a hurry to go to the physician they did not know, they had more confidence with you and asked you their doubts. Then, you realized that they did not understand the things that were being explained to them”*

N.8

Some healthcare professionals detected that **patients were not informed or aware of the need for medication reconciliation when they were discharged**. Therefore, they consider this to be a barrier in communication.

*“[…] I believe that information to patients or relatives should also be improved in some cases, so that when this patient reaches us, it allows us to achieve a better MR. If you come with solid information, what I explain to you will be faster and more efficient”*

P.5

In addition, **the knowledge, practical skills and attitudes of the informal care** giver were identified as fundamental for productive communication about MR.

*“In other cases, it is the caregiver or the family member who brings the pills, puts them in front of him, and the patient does not know what is being taken, and when you ask him they do not know what is being taken of each thing”*

P.2

On the other hand, **the low sociocultural level of patient/informal caregiver**, which is prevalent in certain mainly rural areas, was identified as an element that hinders communication for medication reconciliation.

*“We have a native population of the town that has a slightly lower cultural level, so you have to use a simpler vocabulary with them, which is more effective”*

N.3

*“Sometimes it’s a question that they do not understand our explanations well, I try to write them down in a very detailed way, systematically, but sometimes they do not understand well”*

P.2

##### Facilitator

Two facilitating elements were detected. First, patient/informal caregiver with a **medium/high sociocultural level**, mainly in urban areas, was seen as a facilitator of communication for medication reconciliation.

*“[…] for being a dormitory city, so to speak, for its proximity to Huelva and for being a residential area of villas and residential areas, we have a younger population and with a higher cultural level, so communication with them has other characteristics”*

N.3

**The proximity offered by the primary healthcare field with the provision of home care** was detected as the second element that facilitates communication for correct medication reconciliation at hospital discharge, thus favouring the establishment of a direct relationship.

*“When it comes with the report, if they have not reconciled the medication there, when it comes with the report, we already take the opportunity to do the review and the MR, at the same address or in the consultation”*

P.4

This situation promotes a greater degree of trust in the patient and informal caregiver towards primary healthcare professionals.

## 4. Discussion

This study describes the perceptions of primary healthcare professionals of the barriers and facilitating in elements encountered communication during the medication reconciliation process in the Spanish context. To the best of our knowledge, this is the first study carried out in Spain that identifies the barriers and facilitators of communication for medication reconciliation from the perspective of primary healthcare professionals. The results obtained show, along the lines of other studies, that the challenges in communication can be interlevel, involving transitions between different levels of care [23,29]; among healthcare professionals involved in primary healthcare [22,37,38]; and between healthcare professionals and patients/caregivers [39,40].

Our participants identified communication difficulties with specialized hospital professionals as a communication barrier between hospital care and primary healthcare. They attributed this to the ineffective communication channels and to the difficulties in collaboration between different healthcare professionals. However, they do perceive the existence of technologies, such as computerized medical records, as a facilitator, although it seems that even after two decades of its implementation, they are still seen as insufficient mediums for exchanging information. This feeling is enhanced by the fact that the current tool (Diraya) appears to have not improved this communication exchange, given its structure and the display of varied and inexact information based on the healthcare professional’s profile, which obliges them to look for new and more precise information [13,29,41,42]. This becomes difficult because the healthcare professionals are already overburdened with work and have little or no time available. Lack of time can also influence the quantity and quality of the information that healthcare professionals record in medical records [21,22] which also undoubtedly hinders the proper exchange of such data.

Several authors have pointed out the need to redesign interprofessional work systems to improve patient follow-up [17] In this sense, and taking into account the statements of the participants in our study, this redesign could involve improving computerized clinical histories, and the protocolization of clinical sessions or meetings that can now be carried out via videoconferences and other telematic media with some ease; this has already been proven to be useful [17,22,29]. There is no doubt that, for this to happen, it is also necessary to adjust workloads and include this communication as another care task to be carried out by healthcare professionals. In any case, it would seem to be necessary to include a person-centred care model [43], which allows professionals to focus on the person as a whole and move away from the exclusive focus on their specialty. In fact, different studies have shown how non-integral HC is also one of the difficulties in medication reconciliation [44,45].

In the Spanish context, computerized medical records are only available to hospital care and primary healthcare professionals, but not to pharmacists, whose sole function is to dispense the medication prescribed by physicians of different specialties. Facilitating community pharmacy staff’s access to medical records and encouraging their participation in medication reconciliation processes after discharge could enhance communication and lead to efficient exchange of information; as shown by different studies [46,47], pharmacy staff could become the mediator between hospital and primary healthcare for medication reconciliation.

This observation is context-dependent, and cannot be extrapolated to other countries, particularly the ones with an increasingly aging population and a high prevalence of non-communicable diseases. Moreover, economically developed countries have the advantage of having access to substantial pharmaceutical budgets and an efficient public health system equipped with advanced resources which enables them to respond adequately to the growing health needs of their population [4,48].

In the context where our study is carried out, we find that it is important to have a single record of the patient’s medication which must be used correctly, be updated and accessible to all the healthcare professionals involved, including community pharmacy professionals [3,42,49]. The use of new technologies can also contribute to improving and streamlining communication and care effectiveness [50], especially in the case of multipathological older adults with polypharmacy, thereby enhancing patient safety [15,51,52]. In addition, computerized medical record systems would need to include applications or systems to warn about MR problems. Previous studies have already shown that the lack of a computerized system that alerts healthcare providers of polypharmacy issues in at-risk older adults can be a major barrier to medication reconciliation [22].

Another barrier identified by our particpants was the condition of multipathological and older adults with polypharmacy. Being a patient with polypharmacy and having been treated by more than one medical professional makes medication reconciliation difficult [4]. Some authors describe the transition from hospital care to primary healthcare for these patients as a complex situation which requires establishing protocols prior to discharge and post-discharge follow-up from hospital [53].

Making the patient or family member the mediator between hospital care and primary healthcare is another barrier identified by our participants. This situation was not unusual a few years ago, when there were no computerized medical records and discharge reports were delivered on paper to the patient/caregiver when leaving the hospital; this enabled the primary healthcare professional to be informed of the indications for treatment after hospitalization, when the patient/caregiver would take the report to the physician for consultation. It is striking that current primary healthcare professionals continue to identify this situation as a barrier to communication, despite the existence of tools, such as computerized medical records. However, it does seem that this tool is not properly used or does not have the alert systems that allow primary healthcare professionals to be informed of discharges and changes in the health of their patients, regardless of whether they come for their consultation after hospitalization. It is essential to properly inform patients/caregivers of changes in medication, as some studies show that medication reconciliation improves when this is done correctly [39]. However, it must be a complementary action, and not the primary one, to ensure adequate medication reconciliation.

It is noteworthy that all the healthcare professionals participating in our study highlighted the case management nurse as a key element for medication reconciliation, which is in line with other studies [28]. The main function of case management nurses is ensuring precise coordination and communication between the different levels of care [22]. However, and despite the benefits for interlevel coordination of the actions of the case management nurse, the number of such professionals in primary healthcare centres in Spain is insufficient [54,55]. Therefore, not all older people who are discharged after hospitalization can benefit from the coordination that the case management nurse can provide.

Regarding the barriers to communication between healthcare professionals of the same level of care, our interviewees emphasized, as in other studies, the lack of standardized systems for medication reconciliation, as well as the lack of time [21,22,56]. It therefore seems necessary to generate standardized protocols and processes for communication between primary healthcare professionals, and to reduce workloads in consultation to improve care and free up time for professionals to hold coordination meetings, clinical sessions and other activities that improve the exchange of information and teamwork. In fact, teamwork has been identified in our study, and in others [13], as an important facilitator of medication reconciliation in primary healthcare. Other studies showed that a good relationship between healthcare professionals is key to the continuity of care for patients, especially in older adults with polypharmacy and multipathological condition [39,57].

Regarding communication between primary healthcare professionals and patients/informal caregivers, our participants identified the turnover among healthcare professionals in their centres as a barrier, since it prevents the professionals from acquiring in-depth knowledge on the health status of the individuals and families they serve; this observation coincides with the findings of other studies [58]. Undoubtedly, one of the key elements in primary healthcare is the continuity of care provided over time, which enables healthcare professionals to acquire an exhaustive knowledge of the health conditions of the people they attend to, as well as the changes in them. Therefore, staff turnover is a problem when it comes to following the medication reconciliation process in a structured way.

Another barrier identified by the study participants was related to the characteristics of informal caregivers, as most often they lack knowledge on the skills and attitudes requisite for providing healthcare, as evident from the fact that they sometimes experience difficulties when participating in the conciliation process [17,41]. In this sense, several authors have described useful strategies such as informing patients and caregivers about medications, their training with communication tools and skills or the integration of patients or caregivers as full members of the care team [15,28,40,59]. Other authors have shown that a substantial improvement in the accuracy of information on medication reconciliation at discharge can be achieved when patients participate in this process [17,44].

The medium/high sociocultural level of the patient/caregiver was identified as a facilitator in communication with healthcare professionals, as it improves the effectiveness of the medication reconciliation process. It should be noted that instructions given often do not adjust to the literacy levels or information needs of the patient, which makes it difficult to implement changes [17], so, adapting the information could improve communication between healthcare professionals and patients/caregivers [28,50].

Proximity to the family and awareness of the home environment, with all the elements that this involves, were highlighted by our participants as facilitators of communication between the patient, informal caregiver and the primary healthcare professionals, which coincides with the finding of previous authors [28,60].

### Limitations

This study has certain limitations. As the first and the most exploratory phase of approaching the problem, we decided to focus on medical and nursing professionals since they are, within the Spanish health system, the main actors in the medication reconciliation process. However, our study does not include the perspective of patients, informal caregivers, or other professionals, such as pharmacist. On the other hand, this study represents a localist reality, influenced by a health system that provides universal, free and equitable care, as is the case of the Spanish health system. However, it cannot be extrapolated to other countries that follow different models of health systems or where other professionals, such as pharmacists, are already a part of the medication reconciliation process. We understand that future studies focusing on the perception of patients, family and caregivers, as well as that of other professionals not included in the process, will be important and relevant to enhance medication reconciliation processes.

## 5. Conclusions

The primary healthcare professionals surveyed in this study perceived MR difficulties in interlevel communication, between primary healthcare professionals and between healthcare professionals and patients/caregivers. In general, these difficulties mainly have to do with the adequacy and use of technological tools, the time available, workload and the level of patient’s or family member’s collaboration. It is necessary to improve information technology tools and the quality of computerized medical histories to reduce workloads in consultation, facilitate teamwork among healthcare professionals and generate protocols and strategies that allow the systematization of medication reconciliation processes for older adult patients after hospital discharge. This could help reduce medical errors, particularly in vulnerable population groups, such as older adults, who frequently have multiple pathologies and require polymedication.

As facilitating elements of communication for medication reconciliation, the participants highlighted the use of technologies, such as computerized medical histories, and the protocolization of clinical sessions. New technologies have been proven to improve communication and the effectiveness of care. The participants also emphasized the importance of the role of the case management nurse as key for medication reconciliation and interdisciplinary teamwork, which allows to develop a model of care centred on the person and not on exclusive medical specialty of the professionals. A good relationship between healthcare professionals is needed to improve the continuity of care for patients. In primary healthcare, awareness of the home environment enables the healthcare professional to create a climate of trust to ease communication between healthcare professionals and patients/informal caregivers. Finally, a medium/high sociocultural level in patients/caregivers was seen to facilitate communication in medication reconciliation.

This study demonstrates the need for coordinated, protocolized, effective and multidisciplinary communication among all healthcare professionals involved in the medication reconciliation process at hospital discharge, accommodating patients, family members and caregivers and encouraging their active participation. Only in this way can we ensure quality of care and guarantee patient safety.

It is striking that the barriers and facilitators identified by the Spanish primary healthcare professionals are similar to those described by other studies carried out in other countries with very diverse cultural contexts and health systems. It therefore seems that the professionals’ perceptions of the elements that hinder and facilitate medication reconciliation are very consistent and independent of the country or healthcare environment in which they work. It is undoubtedly something that health system managers should take into account when establishing the necessary measures to improve medication reconciliation.

Finally, we have included the main recommendations to facilitate communication in the medication reconciliation process, extracting of our findings in the following Box 1.

Box 1 **Recommendations to facilitate communication in the medication reconciliation process.**

- Develop a common standardized system for medication reconciliation
- Protocolization of clinical sessions
- Include case management nurses in the medication reconciliation process
- Use up-to-date technologies to save time of the physician, nurse, pharmacist involved in the discharge of patients
- Build collaboration with pharmacists
- Write instructions for patients and caregivers

## Figures and Tables

**Table 1:** Barriers (B) and facilitators (F) of communication in medication reconciliation between levels of care.

B/F	Category		Operational Definition
**B**	Non-existence or ineffectiveness of communication channels		Refers to the existence and functionality that healthcare professionals find when communicating
	Cross-level collaboration		Lack of collaboration on the part of specialists in the hospital field in medication reconciliation
	Lack of relationship with pharmacist		Lack of relationship with pharmacists and valuation of their work in communication. Element that they contemplate as necessary (that they refer to as non-existent or absent) but also referred to as facilitator
	Non-comprehensive care		Unique care within an area of specialization without attending to other pathologies or previous treatments, generating problems in medication reconciliation
	Use of the patient/informal caregivers as a link between levels		Give the patient responsibility for bringing the information relating to discharge to the healthcare professionals of other levels of care
	Chronic multi-pathological patients		Difficulty typical of patients who have multiple pathologies, and the interaction of many and diverse professionals
**F**	Figure of the case management nurse		Case management nurse who serves as liaison between levels. Work of the nurse as mediator between both care spaces
	Technological tools	Professional mailbox	Information through these computer communication resources that informs of, and gives access to, patients and their processes
		Diraya	Communication tool to know the care processes between levels
		Telemedicine	Telematic and internal communication at various levels of specialization for specific consultations of multidisciplinary patients

**Table 2:** Barriers to, and facilitators of, communication on medication reconciliation between healthcare professionals of the same level of care.

B/F	Categories	Operational Definition
**B**	Non-existence/ignorance or lack of use of a common standardized system for medication reconciliation	Non-existence/ignorance or lack of use of common standardized systems for medication reconciliation. Each healthcare professional acts differently.
	Lack of time for communication between healthcare professionals	Lack of time for communication between nurse and physician, and to manage cases of highly complex patients in medication reconciliation.
**F**	Protocols	Knowledge and use of action protocols, where the interventions and the form of action between healthcare professionals involved in medication reconciliation are unified.
	Close relationship/Teamwork	Close relationship between the two healthcare professionals involved in medication reconciliation. Fluid communication and strategies aimed at responding to the same problem.

**Table 3:** Barriers to, and facilitators of, communication on medication reconciliation process between primary healthcare professionals and patients/informal caregivers.

B/F	Categories	Operational Definition
**B**	Geographical distribution of the health centre (urban or rural)	The number of patients to be treated, according to the characteristics of the geographical space in which they are treated.
	Unstable job conditions of the staff	Lack of contact, knowledge and fluid and effective communication caused by staff turnover. They do not know the patients and their circumstances.
	Patient is unaware of the need for medication reconciliation.	The patient in hospital care is not made to participate in the need for medication reconciliation at discharge.
	Informal caregiver characteristics: knowledge, skill and attitudes	Lack of knowledge and skills of caregivers in the communication process. It hinders communication and transfer of information from the healthcare professional to these mediators.
	Low sociocultural level	Difficulty observed in communication for medication reconciliation with people of low sociocultural level.
**F**	Medium/high sociocultural level	The medium/high sociocultural level facilitates and improves the effectiveness of communication for medication reconciliation.
	Proximity of the primary healthcare centre to the home space	Knowledge acquired by the healthcare professional of the patient and family environment, and knowledge of their communication channels and intra-family relationship.

## Data Availability

Data will be made available upon request by the Corresponding author.

## References

[1] World Health Organization (2017). Medication Without Harm. WHO Global Patient Safety Challenge.

[2] Alanazi F.K., Sim J., Lapkin S. (2022). Systematic review: Nurses’ safety attitudes and their impact on patient outcomes in acute-care hospitals. Nurs. Open.

[3] Stark H.E., Graudins L.V., McGuire T.M., Lee C.Y.Y., Duguid M.J. (2020). Implementing a sustainable medication reconciliation process in Australian hospitals: The World Health Organization High 5s project. Res. Soc. Adm. Pharm..

[4] Jakovljevic M., Jakab M., Gerdtham U., McDaid D., Ogura S., Varavikova E., Merrick J., Adany R., Okunade A., Getzen T.E. (2019). Comparative financing analysis and political economy of noncommunicable diseases. J. Med. Econ..

[5] Jakovljevic M., Yamada T., Grujic D. (2023). Editorial: Role of health economic data in policy making and reimbursement of new medical technologies, Volume II. Front. Public Health.

[6] Masnoon N., Shakib S., Kalisch-Ellett L., Caughey G.E. (2017). What is polypharmacy? A systematic review of definitions. BMC Geriatr..

[7] Mejía G., Saiz-Rodríguez M., Gómez de Olea B., Ochoa D., Abad-Santos F. (2020). Urgent Hospital Admissions Caused by Adverse Drug Reactions and Medication Errors—A Population-Based Study in Spain. Front. Pharmacol..

[8] Al Rihani S.B., Deodhar M., Darakjian L.I., Dow P., Smith M.K., Bikmetov R., Turgeon J., Michaud V. (2021). Quantifying Anticholinergic Burden and Sedative Load in Older Adults with Polypharmacy: A Systematic Review of Risk Scales and Models. Drugs Aging.

[9] Asadi H., Habibi Soola A., Iranpour S. (2022). Evaluation of the Relationship Between Frailty and Polypharmacy in the Elderly Referred to the Emergency Departments of Ardabil 2019. Salmand Iran. J. Ageing.

[10] Stolldorf D.P., Ridner S.H., Vogus T.J., Roumie C.L., Schnipper J.L., Dietrich M.S., Schlundt D.G., Kripalani S. (2021). Implementation strategies in the context of medication reconciliation: A qualitative study. Implement. Sci. Commun..

[11] Tobiano G., Chaboyer W., Teasdale T., Raleigh R., Manias E. (2019). Patient engagement in admission and discharge medication communication: A systematic mixed studies review. Int. J. Nurs. Stud..

[12] Alqenae F.A., Steinke D., Keers R.N. (2020). Prevalence and Nature of Medication Errors and Medication-Related Harm Following Discharge from Hospital to Community Settings: A Systematic Review. Drug Saf..

[13] Latimer S., Hewitt J., de Wet C., Teasdale T., Gillespie B.M. (2023). Medication reconciliation at hospital discharge: A qualitative exploration of acute care nurses’ perceptions of their roles and responsibilities. J. Clin. Nurs..

[14] Latimer S., Hewitt J., Teasdale T., de Wet C., Gillespie B.M. (2020). The accuracy, completeness and timeliness of discharge medication information and implementing medication reconciliation: A cross-sectional survey of general practitioners. Aust. J. Gen. Pract..

[15] van der Nat D.J., Huiskes V.J.B., Taks M., van den Bemt B.J.F., van Onzenoort H.A.W. (2022). Barriers and facilitators for the usage of a personal health record for medication reconciliation: A qualitative study among patients. Br. J. Clin. Pharmacol..

[16] Dias Fernandes B., Fernandes Almeida P.H.R., Foppa A.A., Tavares Sousa C., Rocha Ayres L., Chemello C. (2020). Pharmacist-led medication reconciliation at patient discharge: A scoping review. Res. Soc. Adm. Pharm..

[17] Hannum S.M., Abebe E., Xiao Y., Brown R., Peña I.M., Gurses A.P. (2021). Engineering care transitions: Clinician perceptions of barriers to safe medication management during transitions of patient care. Appl. Ergon..

[18] Al Anazi A. (2021). Medication reconciliation process: Assessing value, adoption, and the potential of information technology from pharmacists’ perspective. Health Inform. J..

[19] Institute of Health Improvement (2008). How-to Guide: Prevent Adverse Drug Events (Medication Reconciliation).

[20] Considine J., Berry D., Sprogis S.K., Newnham E., Fox K., Darzins P., Rawson H., Street M. (2020). Understanding the patient experience of early unplanned hospital readmission following acute care discharge: A qualitative descriptive study. BMJ Open.

[21] Gionfriddo M.R., Duboski V., Middernacht A., Kern M.S., Graham J., Wright E.A. (2021). A mixed methods evaluation of medication reconciliation in the primary care setting. PLoS ONE.

[22] Sun W., Tahsin F., Barakat-Haddad C., Turner J.P., Haughian C.R., Abbass-Dick J. (2019). Exploration of home care nurse’s experiences in deprescribing of medications: A qualitative descriptive study. BMJ Open.

[23] Ozavci G., Bucknall T., Woodward-Kron R., Hughes C., Jorm C., Joseph K., Manias E. (2021). A systematic review of older patients’ experiences and perceptions of communication about managing medication across transitions of care. Res. Soc. Adm. Pharm..

[24] Gionfriddo M.R., Hu Y., Maddineni B., Kern M., Hayduk V., Kaledas W.R., Elder N., Border J., Frusciante K., Kobylinski M. (2022). Evaluation of a Web-Based Medication Reconciliation Application Within a Primary Care Setting: Cluster-Randomized Controlled Trial. JMIR Form. Res..

[25] Leal-David M.H., Martínez-Riera J.R., Herraiz-Mallebrera A., Lima da Costa M.F. (2020). Case management nurse in Spain: Facing the challenge of chronicity through a comprehensive practice. Cien. Saude. Colet..

[26] Rangachari P., Dellsperger K.C., Fallaw D., Davis I., Sumner M., Ray W., Fiedler S., Nguyen T., Rethemeyer R.K. (2019). A Mixed-Method Study of Practitioners’ Perspectives on Issues Related to EHR Medication Reconciliation at a Health System. Qual. Manag. Health Care.

[27] Rojas-Ocaña M.J., García-Navarro E.B., García-Navarro S., Macías-Colorado M.E., Baz-Montero S.M., Araujo-Hernández M. (2022). Influence of the COVID-19 Pandemic on Medication Reconciliation in Frail Elderly People at Hospital Discharge: Perception of Healthcare Professionals. Int. J. Environ. Res. Public Health.

[28] Macías-Colorado M.E., Rodríguez-Pérez M., Rojas-Ocaña M.J., Teresa-Morales C. (2021). Communication on Safe Caregiving between Community Nurse Case Managers and Family Caregivers. Healthcare.

[29] Syyrilä T., Vehviläinen-Julkunen K., Härkänen M. (2021). Healthcare professionals’ perceptions on medication communication challenges and solutions–text mining and manual content analysis-cross-sectional study. BMC Health Serv. Res..

[30] Tong A., Sainsbury P., Craig J. (2007). Consolidated criteria for reporting qualitative research (COREQ): A 32-item checklist for interviews and focus groups. Int. J. Qual. Health Care.

[31] (1998). Andalusian Regional Governement, Law 2/1998, 15 of June, of Health in Andalusia. https://www.boe.es/eli/es-an/l/1998/06/15/2/con.

[32] Andalusian Health System (2012). Typology of Health Centers in the Analytical Accounting System.

[33] Naderifar M., Goli H., Ghaljaie F. (2017). Snowball Sampling: A Purposeful Method of Sampling in Qualitative Research. Strides Dev. Med. Educ..

[34] Sandelowski M. (1995). Sample size in qualitative research. Res. Nurs. Health.

[35] Graneheim U.H., Lundman B. (2004). Qualitative content analysis in nursing research: Concepts, procedures and measures to achieve trustworthiness. Nurse Educ. Today.

[36] Lincoln Y., Guba E. (1985). Naturaliztic Inquiry.

[37] Manias E., Cranswick N., Newall F., Rosenfeld E., Weiner C., Williams A., Wong I.C., Borrott N., Lai J., Kinney S. (2019). Medication error trends and effects of person-related, environment-related and communication-related factors on medication errors in a paediatric hospital. J. Paediatr. Child Health.

[38] Mendizabal Olaizola A., Valverde E., Goienetxea E., Oñatibia A., Ezcurra M. (2019). Reconciliation of medication in coordination between primary care professionals and the community pharmacy. Int. J. Integr. Care.

[39] Wheeler A.J., Scahill S., Hopcroft D., Stapleton H. (2018). Reducing medication errors at transitions of care is everyone’s business. Aust. Prescr..

[40] Bucknall T., Fossum M., Hutchinson A.M., Botti M., Considine J., Dunning T., Hughes L., Weir-Phyland J., Digby R., Manias E. (2019). Nurses’ decision-making, practices and perceptions of patient involvement in medication administration in an acute hospital setting. J. Adv. Nurs..

[41] Pinelli V., Stuckey H.L., Gonzalo J.D. (2017). Exploring challenges in the patient’s discharge process from the internal medicine service: A qualitative study of patients’ and providers’ perceptions. J. Interprofessional Care.

[42] Waldron C., Cahill J., Cromie S., Delaney T., Kennelly S.P., Pevnick J.M., Grimes T. (2021). Personal Electronic Records of Medications (PERMs) for medication reconciliation at care transitions: A rapid realist review. BMC Med. Inform. Decis. Mak..

[43] Errasti-Ibarrondo B., Choperena A., Wilson D.M. (2022). Reading and reflecting on experiential accounts of hospital patients to foster a person-centered care approach: A novel educational method. Teach. Learn. Nurs..

[44] Daliri S., Bekker C.L., Buurman B.M., Scholte op Reimer W.J.M., van den Bemt B.J.F., Karapinar-Çarkit F. (2019). Barriers and facilitators with medication use during the transition from hospital to home: A qualitative study among patients. BMC Health Serv. Res..

[45] Penm J., Vaillancourt R., Pouliot A. (2019). Defining and identifying concepts of medication reconciliation: An international pharmacy perspective. Res. Soc. Adm. Pharm..

[46] Fournier R., Kachachi S., Mouchoux C., Gervais F. (2022). From medication reconciliation to shared medication review: Pilot study integrating support for community pharmacists within a pharmaceutical care pathway. Ann. Pharm. Fr..

[47] Marinovic I., Bacic Vrca V., Samardzic I., Marusic S., Grgurevic I., Papic I., Grgurevic D., Brkic M., Jambrek N., Mesaric J. (2021). Impact of an integrated medication reconciliation model led by a hospital clinical pharmacist on the reduction of post-discharge unintentional discrepancies. J. Clin. Pharm. Ther..

[48] Cerda A.A., García L.Y., Rivera-Arroyo J., Riquelme A., Teixeira J.P., Jakovljevic M. (2022). Comparison of the healthcare system of Chile and Brazil: Strengths, inefficiencies, and expenditures. Cost Eff. Resour. Alloc..

[49] Welk B., Killin L., Reid J.N., Anderson K.K., Shariff S.Z., Appleton A., Kearns G., Garg A.X. (2021). Effect of electronic medication reconciliation at the time of hospital discharge on inappropriate medication use in the community: An interrupted time-series analysis. CMAJ Open.

[50] Mitchell S., Laurens V., Weigel G., Hirschman K., Scott A., Nguyen H. (2018). Care transitions from patient and caregiver perspectives. Ann. Fam. Med..

[51] Astier-Peña M.P., Torijano-Casalengua M.L., Olivera-Cañadas G. (2016). Setting priorities for patient safety in Primary Care. Aten. Primaria..

[52] Kattel S., Manning D.M., Erwin P.J., Wood H., Kashiwagi D.T., Murad M.H. (2020). Information Transfer at Hospital Discharge: A Systematic Review. J. Patient Saf..

[53] Allen J., Hutchinson A.M., Brown R., Livingston P.M. (2018). User experience and care for older people transitioning from hospital to home: Patients’ and carers’ perspectives. Health Expect..

[54] Miguélez-Chamorro A., Casado-Mora M.I., Company-Sancho M.C., Balboa-Blanco E., Font-Oliver M.A., Román-Medina Isabel I. (2019). Advanced practice in case management: An essential element in the new complex chronicity care model. Enfermería Clínica.

[55] Duarte-Climents G., Sánchez-Gómez M.B., Rodríguez-Gómez J., Rodríguez-Álvarez C., Sierra-López A., Aguirre-Jaime A., Gómez-Salgado J. (2019). Impact of the Case Management Model through Community Liaison Nurses. Int. J. Environ. Res. Public Health.

[56] Bosque D., Forbes S., Ward E.N., Delaney J., Meyers G.T. (2021). Reconciliation and Disposal of Oral Medication: Creating a Safe Process for Clinical Research Personnel. Clin. J. Oncol. Nurs..

[57] Lane-Fall M.B., Pascual J.L., Peifer H.G., Di Taranti L.J., Collard M.L., Jablonski J., Gutsche J.T., Halpern S.D., Barg F.K., Fleisher L.A. (2020). A Partially Structured Postoperative Handoff Protocol Improves Communication in 2 Mixed Surgical Intensive Care Units: Findings From the Handoffs and Transitions in Critical Care (HATRICC) Prospective Cohort Study. Ann. Surg..

[58] Aires-Moreno G.T., Silvestre C.C., Araujo D., Matos V.T.G., Marcon de Oliveira V., Ferreira C.M., Vasconcelos-Pereira E.F., Lira A.R.P., Chemello C., Oliveira L.M.S. (2020). Perceptions of nurses, pharmacists and physicians about medication reconciliation: A multicenter study. Saudi. Pharm. J..

[59] Kim J.M., Suarez-Cuervo C., Berger Z., Lee J., Gayleard J., Rosenberg C., Nagy N., Weeks K., Dy S. (2018). Evaluation of Patient and Family Engagement Strategies to Improve Medication Safety. Patient.

[60] Moral-Fernández L., Frías-Osuna A., Moreno-Cámara S., Palomino-Moral P.A., del-Pino-Casado R. (2018). The first moments of the carer: The process of becoming a caregiver of a dependent elderly relative. Atención Primaria.

